# What Do We Mean by 'Community Resilience'? A Systematic Literature Review of How It Is Defined in the Literature

**DOI:** 10.1371/currents.dis.db775aff25efc5ac4f0660ad9c9f7db2

**Published:** 2017-02-01

**Authors:** Sonny S. Patel, M. Brooke Rogers, Richard Amlôt, G. James Rubin

**Affiliations:** Department of Psychological Medicine, King's College London, London, United Kingdom; Department of Global Health and Population, Harvard T.H. Chan School of Public Health, Boston, Massachusetts, USA; Department of War Studies, King's College London, London, United Kingdom; Emergency Response Department, Public Health England, Salisbury, United Kingdom; Department of Psychological Medicine, King's College London, London, United Kingdom

**Keywords:** community resilience, disaster, emergency response, extreme events, governance, policy, preparedness, Public Health, Resiliency

## Abstract

**Background:**

Government, industry and charitable organisations have an increasing focus on programs intended to support community resilience to disasters. But has consensus been reached as to what defines 'community resilience' and what its core characteristics are?

**Methods:**

We undertook a systematic literature review of definitions of community resilience related to disasters. We conducted an inductive thematic analysis of the definitions and descriptions that we identified, in order to determine the proposed characteristics of community resilience prior to, during and after a disaster.

**Results:**

We identified 80 relevant papers. There was no evidence of a common, agreed definition of community resilience. In spite of this, evidence was found of nine core elements of community resilience that were common among the definitions. The core elements were: local knowledge, community networks and relationships, communication, health, governance and leadership, resources, economic investment, preparedness, and mental outlook. Within these core elements, we identified 19 sub-elements linked to community resilience.

**Conclusion:**

Our findings show that community resilience remains an amorphous concept that is understood and applied differently by different research groups. Yet in spite of the differences in conception and application, there are well-understood elements that are widely proposed as important for a resilient community. A focus on these individual elements may be more productive than attempting to define and study community resilience as a distinct concept.

## Introduction

The ability to operationalize the concept of ‘community resilience’ against a disaster is highly sought after by disaster-response professionals, government officials, and academics. With the effects of climate change and demographic movements into large cities, disasters are occurring more frequently and in many cases with higher intensity than in previous years^1^^,^^2^^,^^3^ When disasters strike, governments and aid organizations are not always in a position to help communities immediately. For example, in Canada, the official emergency preparedness guide urges families to “*be prepared to take care of yourself and your family for a minimum of 72 hours*” if an emergency happens in their community because “*it may take emergency workers some time to reach you* (p. 3)”^4^. In America, States such as New Hampshire and California have created public awareness campaigns and websites to increase disaster preparedness activities, such as assembling household emergency kits^5^^,^^6^. Even when governments and organizations can help, their resources are often stretched. Speaking to the press in 2014, Valerie Amos, Under-Secretary-General for Humanitarian Affairs and Emergency Relief Coordinator at the United Nations, noted that “*the world’s collective response capacity and resources are being stretched to the limit*” by the number of disasters that had occurred in 2013^7^. Because of these issues, recent attention has focused on how best to help communities to help themselves, with a concomitant focus on understanding what factors contribute to making a community resilient to disasters.

This focus has been accompanied by a change in rhetoric from government, industry and charitable organisations from discussing ‘disaster vulnerability’ to ‘disaster resilience,’ which is “*viewed as a more proactive and positive expression of community engagement with natural hazard reduction* (p. 598)”^8^. The term ‘disaster’ is defined by UNISDR as “a serious disruption of the functioning of a community or a society involving widespread human, material, economic or environmental losses and impacts, which exceeds the ability of the affected community or society to cope using its own resources”^9^, while a ‘community’ can be broadly defined as a constituent population such as a neighbourhood, town, or city. For example, the Rockefeller Foundation and Arup International Development recently created the City Resilience Framework^10^ to aid with evidence-based policy, to reduce the disaster risk in cities and to identify functions of what makes a resilient city. On an international scale, substantial improvements have been made with shifting policies and activities related to disaster risk reduction. Recent efforts by the United Nations have led to the creation of the Sendai Framework for Disaster Risk Reduction 2015-2030, which is built upon the Hyogo Framework for Action 2005-2015. This recent international treaty emphasises specific outcomes and priorities related to disaster risk reduction to be achieved by 2030, such as, “the substantial reduction of disaster risk and losses in lives, livelihoods and health in the economic, physical, social, cultural and environmental assess of persons, businesses, communities and countries (p. 12)"^11^.

The concept of ‘community resilience’ is almost invariably viewed as positive, being associated with increasing local capacity^12^^,^^13^^,^^14^, social support^15^^,^^16^ and resources^17^^,^^18^, and decreasing risks^19^^,^^20^^,^^21^, miscommunication^22^^,^^23^^,^^24^ and trauma^25^^,^^26^^,^^27^. Yet consensus as to what community resilience is, how it should be defined and what its core characteristics are does not appear to have been reached, with mixed definitions appearing in the scientific literature, policies and practice^28^^,^^29^^,^^30^. This confusion is troubling. The way we define community resilience affects how we attempt to measure and enhance it. For example, the Communities Advancing Resilience Toolkit (CART) describes a resilient community as one that “*has the ability to transform the environment through deliberate, collective action” and “requires that the community as a whole must cope effectively with and learn from adversity* (p. 1)"^31^ and as such suggests the measurement of community resilience requires the measurement of specific constructs such as ‘transformative potential,’ ‘connection and caring,’ ‘resources’ and ‘disaster management’ in order to identify areas of weakness. In contrast, the Conjoint Community Resiliency Assessment Measure (CCRAM) defines community resilience as “*the community’s ability to withstand crises or disruptions* (p. 1732)”^32^ and emphasises variables relating to leadership, collective efficacy, place attachment, preparedness, and social trust^32^. Identical communities may score very differently on these two measures of what is supposed to be the same phenomenon.

We conducted a systematic review of definitions of community resilience as it relates to disasters, in order to identify the range of definitions of community resilience present in the literature and to identify the range of constituent elements of community resilience that have been proposed.

## Methods

We initially completed a systematic literature search with no start date and a publication cut-off date of October 2013 for scientific peer-reviewed articles and of January 2014 for grey literature. Peer reviewed papers were found through searches of MEDLINE and PsycInfo, which were searched from inception. The search used keywords based on the stem words of resilience AND disaster AND definition (Table 1). We subsequently updated the search for peer reviewed papers using the same strategy for papers published up to May 2015.

**Table 1. Keywords Used for Database Searches:** 

Used Keywords for MEDLINE Searches
resilen* AND disast* AND defin*
(Community resilience or neighbourhood resilience or neighbourhood resilience or social resilience or social capital) AND (disaster or flood* or volcano or hurricane? Or Chernobyl or Fukushima or earthquake) AND (definition?) or (framework or taxonomy)
Resilien* AND (defin* or fram*) AND (disaster or flood* or volcano or hurricane? or Chernobyl or Fukushima or earthquake)
Used Keywords for Google Searches::
Community resilience AND disaster AND (definition or framework)
Resilience AND disaster AND (definition or framework)

Grey literature searches using the keywords listed in Table 1 were applied using the search engine Google. The grey literature search was initially undertaken in October 2013, and updated in January 2014. Due to the volume of pages found in this search, we only reviewed the first 60 unique links. If two or more links came from the same main website, this was considered one unique link. Within the first 60 unique links, we extracted any publications that appeared relevant for further examination (e.g. annual reports or educational handouts). In addition to the electronic searches, we reviewed the references cited in all papers and reports.

We included papers if they were written in English, were on the topic of disasters and had a description or discernible definition of community resilience. We defined ‘disasters’ as meeting the UNISDR definition of disaster^9^ and including acts of violence such as war and terrorism as well as natural disasters. Acts of violence such as war and terrorism were included in this review because there are strong similarities between aspects of community resilience to terrorism and war and aspects of community resilience to disasters. We excluded papers discussing epidemics, for example the HIV/AIDS epidemic in Southern Africa^33^. Publications that included resilience definitions relating to individuals^26^^,^^34^^,^^35^^,^^36^^,^^37^^,^^38^, children^39^^,^^40^^,^^41^^,^^42^^,^^43^^,^^44^ or hospital-based systems^45^^,^^46^^,^^47^ were not included, unless the authors also related the definition to a community as a whole. We acknowledged the ambiguity inherent in the definition of ‘community’ and accepted a publication if it attempted to describe resilience as a population-based concept. If publications suggested a wider theory, which included community resilience as part of the theory, we accepted it, but only if these publications included a description or definition of community resilience in respect to the theory (e.g. Zakour and Gillespie^48^).

We screened publications by reading the abstract or summary to remove duplicates, non-English reports, or papers that did not discuss resilience in a community setting in relation to a disaster. If an abstract or summary was not provided, we electronically searched the document for references where the term “resilience” was mentioned and read the relevant section to see if resilience was discussed in a community setting and in relation to a disaster. Potentially relevant publications were read in full.

We created an evidence table, extracting the following from each selected publication: study description, direct quotation of definition, elements of definition as described or inferred by study and any measurable examples given by study. Using QSR International's NVivo 10 qualitative data analysis software, we carried out an inductive thematic analysis to compile a list of common elements within the concept of community resilience based on the definitions found in the literature^49^^,^^50^. This list was determined by the descriptions used in the definitions, as we categorized them into relevant overarching themes (elements) based on their similarities. The final number of elements was reached when no new theme could be uniquely supported or reduced into other existing elements. The same approach was used for the sub-elements within each element.

## Results

Figure 1 shows the results of the search strategy. Overall, we identified 615 publications. Of these, 578 papers were found through MEDLINE and PsychInfo and 37 were from the grey literature.

**Flow diagram of selection process:**
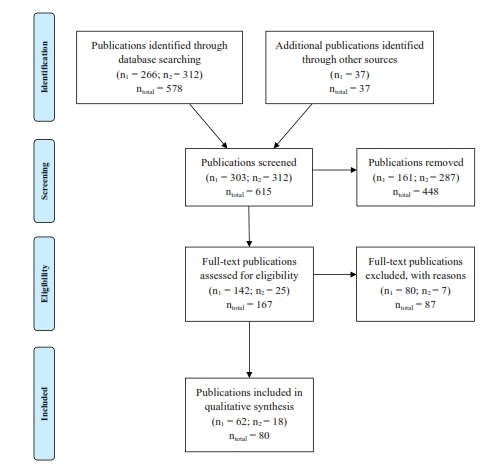
This figure shows the method of selecting a publication to be reviewed. The numbers related to the first search with publication cutoff date of October 2013 for scientific peer-reviewed articles and January 2014 for grey-literature are listed with n_1_, and n_2_ is numbers related to the second search with publication cutoff date of May 2015. The overall numbers are listed as n_total_.

**Definitions of community resilience**

From 62 publications found in the first search, we identified 57 unique definitions of community resilience as it applies to disasters. From the second search, 18 publications were added along with their respective definitions of community resilience. The unique definitions can be found in on-line supplementary Table 1.

Three general types of definition were found: 1) ‘process’ definitions (i.e. an ongoing process of change and adaptation); 2) ‘absence of adverse effect’ definitions (i.e. an ability to maintain stable functioning); and 3) ‘range of attributes’ definitions (i.e. a broad collection of response-related abilities). More recent studies tended to adopt the first type of definition. For example, in an article discussing response enhancements to chemical, biological, radiological and nuclear terrorism, Lemyre and colleagues^51^ called the construct of resilience “*a process or the attainment of positive outcomes at the individual, family, and community levels despite adversity (e.g., natural disaster, terrorist attack)* (pg. 319).” In a review paper on community resilience, Norris and colleagues^52^ defined community resilience as “*a process linking a set of networked adaptive capacities to a positive trajectory of functioning and adaptation in constituent populations after a disturbance* (p. 131).” Citing publications by Paton and colleagues^53^ and Norris and colleagues^52^, Cox and Perry^54^ defined community resilience as “*a reflection of people’s shared and unique capacities to manage and adaptively respond to the extraordinary demands on resources and the losses associated with disasters*” (p. 396). Furthermore, in a recent literature review on resilience, Castleden and colleagues^55^ defined community resilience as “*a capability (or process) of a community adapting and functioning in the face of disturbance* (p.370).”

The ‘absence of adverse effect’ definitions used the desired outcome of ‘maintaining stable functioning’ as their basis. Bonanno^25^ examined the evidence on resilience based on loss and trauma among adults and defined it as an ability of adults to “*maintain relatively stable, healthy levels of psychological and physical functioning* (p.20).” Contrasting to the first type of definition, Gibson^56^ stated in a paper exploring the 2009 Victorian Bushfire in Australia that “*…resilience is not a process, it is not a management system standard, nor is it a consulting product. Resilience is a demonstrable outcome of an organization’s capability to cope with uncertainty and change in an often volatile environment. Resilience is thus a product of an organization’s capabilities interacting with its environment* (p. 246).”

This notion of community resilience as an outcome was adapted by others who noted the importance of specifically identifying and strengthening abilities in a community, creating a third type of definition: the ‘range of positive attributes’ definition. An example of these definitions can be found in a publication by the UK Cabinet Office^57^ which defined community resilience, as “*communities and individuals harnessing local resources and expertise to help themselves in an emergency, in a way that complements the response of the emergency services* (p. 11)”. This report suggests that primarily, community resilience has to do with having a responsive and collective action of local support to help the community after an incident. Research carried out by Coles and Buckle^18^ into community resilience in Australia and the United Kingdom, led them to see resilience as “*a multi-dimensional attribute that in its different forms contributes in various but equally important ways to disaster recovery* (p. 6)." Their definition of community resilience is inferred from their publication in 2004 and summarized in a review article by Norris and colleagues^52^ as “*a community’s capacities, skills and knowledge that allows the community to participate fully in the recovery from disasters* (p. 129).” Moreover, by carrying out an epidemiological study of violence, injury, and resilience among the low-income communities in Western Cape of South Africa, Ahmed and colleagues^58^ defined community resilience by the features of a community. They found the following as key defining dimensions of community resilience specific to their study: “*household relationships, levels of education and literacy, employment-seeking behaviours, social support networks, ability to seek support services, sense of communal safety and hope, and physical security measures* (p. 393)"^58^.

Additionally, definitions exist that blend one or more of these general definition types. In a recent review on assessment models and tools of community disaster resilience, Ostadtaghizadeh and colleagues^59^ produced a definition of community resilience now used by the United Nations International Strategy for Disaster Reduction: the “*ability of a system, community, or society exposed to hazards to resist, absorb, accommodate to and recover from the effects of a hazard in a timely and efficient manner including through the preservation and restoration of its essential basic structures and functions* (p. 3).” This definition blended the general types of ‘absence of adverse effect’ definition and ‘range of positive attributes’ definition. Even more broadly, Pfefferbaum and colleagues^60^ generally defined resilience “*as an attribute (e.g., ability, capacity), a process, and/or an outcome associated with successful adaption to, and recovery from adversity*” and that it “*differs depending on context and purpose* (p. 241-242).”

Community resilience was therefore found to be an amorphous concept that was understood and applied differently by different research groups. In essence, depending on one’s stance, community resilience can either be seen as an ongoing process of adaptation, the simple absence of negative effects, the presence of a range of positive attributes, or a mixture of all three. However, common elements of community resilience were found among the literature based on these various definitions.

**Elements of community resilience**

Within the definitions, we identified nine main elements and 19 sub-elements that have been proposed as important within the concept of community resilience, shown in supplementary Table 2. These are described below with the main element listed in bold and sub-elements in italics. Additionally, some of the sub-elements could feasibly have been placed within other elements. We decided where to place each sub-element based on the emphasis given by the original authors.

A comparison of the elements found in widely cited reviews, models and measurements of community resilience is given in supplementary Table 3. No existing models or measurements incorporated all of the elements and sub-elements identified.

**Local knowledge**: The effects of a disaster, whether short-term or long-term, could be mitigated if a community understands its existing vulnerabilities. These vulnerabilities, if addressed prior to a disaster, are believed to build resilience within a community. For example, Kennedy and colleagues^12^ emphasised the importance of having a community assess and understand their own vulnerabilities. Three sub-elements were found within this. The first was the *factual knowledge base* of the community. Defined as the information, education and experience acquired in relation to a disaster, factual knowledge included specific learned information related to a disaster or disaster preparedness, such as knowledge about first aid^55^, and other issues translatable to disaster preparedness, mitigation, response, and recovery^61^.

*Training and education* was a second sub-element. For example, Moore and colleagues^24^ found exemplary practices in community education, such as including public disaster education within routine education curricula, having early warning and public communications, partnering with the media for public education and risk communication, and communicating with affected populations via newsletters. Additionally, Moore and colleagues^24^ proposed practice to be an element of community resilience with activities such as community training and exercises proposed to build local knowledge and capacity. Effective training and education should lead to learning^62^. For example, Cutter et al 8^,^^63^ emphasise the importance of learning how to respond effectively to an emergency.

The third sub-element found was *collective efficacy and empowerment*. This was defined as a community’s shared belief of its ability to overcome potential hardships caused by a disaster, for example through self-reliance. What the community knows and understands about their own processes to endure and respond to a disaster can be crucial in relief efforts especially if a community is dependent upon its own resources. This was described by Chandra and colleagues^22^, who suggested the importance of strengthening: 1) personal and community preparedness; 2) civic responsibility; 3) effective bystander responses; and 4) self-and community-reliance.

**Community networks and relationships**: Positive effects on a community and its members can occur during a crisis when its members are well *connected* and form a *cohesive* whole. The connectedness of a community, sometimes called its ‘social network’, was defined by the linkages within a community. Creating links among community members based on social relationships^31^^,^^60^^,^^61^ and/or between communities^62^ were examples of connectedness. The cohesion of a community is based on the nature of these links, typically described as weak or strong ties. Several factors which determine the strength of a tie, including trust^13^^,^^16^^,^^50^^,^^53^ and shared values^16^ ^17^^,^^31^^,^^63^ might be relevant to enhanced community resilience. The connectedness of the networks and their cohesion were also discussed as important aspects of social capital, which conceptually focuses on bonding, bridging, and linking^16^.

**Communication: ***Effective communication* was seen as an important by most authors. Different ways to achieve effective communication were highlighted by different authors, however. For example, Norris and colleagues^52^ defined communication as “*the creation of common meanings and understandings and the provision of opportunities for members to articulate needs, views, and attitudes* (p. 140).” The authors interpreted effective communication as having occurred if the community used common meanings for all to understand and if the community provided opportunities for open dialogue. Another aspect of effective communication was the establishment of appropriate communication infrastructure that could be coordinated in a pre- or post-disaster setting. Chandra and colleagues^22^ noted that “*strong communication networks are critical for resilience* (p. 20)” and suggested that networks should have “*diversity of mode and content*,” for example through the use of social media to support and promote emergency messages, preferably using a trusted and established source of information.

Both before and after a disaster, *risk communication* should provide accurate information about possible threats. This was mentioned in several papers, especially by Chandra and colleagues^22^ where the authors suggested training “*partners and lay health advisors in proper risk communication techniques* (p. 20).” Another suggestion was that government officials “*should consider community norms and the range of individual beliefs* (p. 21)” when crafting risk messages^22^ to ensure that the messages address the expectations of community members and are placed in an appropriate social context to help the public understand them^22^^,^^24^. Castleden and colleagues^55^ proposed integrating such steps into a detailed vulnerability analysis and subsequent community awareness initiatives.

During a disaster, *crisis communication* should provide up-to-date information to community members about the ongoing impact and relief efforts. For example, Ganor and Ben-Lavy^64^ found that flow of information in real time was important for relief efforts while Dawes and colleagues^13^ identified open communication during an incident as important to crisis communication. Both issues can also be seen in a community and media approach to community resilience by Houston and colleagues^65^. Their approach focused on the interactions between the strategic communication processes, such as community competence and community narratives; community relationships, such as social capital and media relations; community attributes, such as equality and diversity; and community systems and resources, such as communication infrastructure and traditional and social media. Effective response coordination within a community was found to rely on the communication between agencies, organizations, and community members^55^. Furthermore, Cox and Hamlen^61^ suggested the importance of communications during the crisis, specifically infrastructure and technology.

**Health: **The pre-existing health of a community and delivery of health services after a disaster are important for community resilience. Understanding and addressing health vulnerabilities can build resilience before a disaster and mitigate long-term issues after a disaster. One main sub-element identified within this was *health services*. Health services can be disrupted during a disaster. For example, mass casualties and blackouts could cause problems for a small community healthcare facility. Rego and Mehta^21^ suggested “*building hospitals to higher standards of disaster resilience* (p. 34).” This approach was adopted by the Asian Disaster Preparedness Center’s (ADPC), improvement to healthcare services through training and capacity-building at the hospital and facility level to handle mass casualties. For example, ADPC assisted in integrating these standards to a hospital in Kachchh, Gujarat by replacing the hospital building, which collapsed during the Gujarat earthquake of 2001 and claimed 176 lives, to a building with earthquake-resistant technology^21^.

The delivery and quality of care for *physical* and* mental health* issues were also identified as important sub-elements in health. When disasters hit, casualties with physical injuries must be treated quickly and with a high quality of care. A suggestion by Chandra and colleagues^22^ overlapped with other elements, such as resources, economic investment and preparedness. They suggested a plan for having post-health incident housing, especially for low-income individuals, in order to help restore physical health and livelihoods. Having such a capability would ensure a high level of quality and delivery of care post-disaster.

The mental health of a community can be affected long after a disaster, with the immediate trauma and more chronic secondary stressors resulting in a range of disorders including post-traumatic stress disorder, anxiety and depression in a significant minority of individuals^64^^,^^66^. Providing “*‘psychological first aid’ or other early psychological or behavioral health interventions after a disaster* (p. 15)” were examples of ways in which a community might reduce psychological distress^22^. Additionally, bolstering psychological wellness through public health communication campaigns was another example of ways to bolster resilience^22^.

**Governance/ leadership: **Governance and leadership shape how communities handle crises. We found two sub-elements within governance and leadership: *infrastructure and services*, and *public involvement and support*. For a community’s infrastructure and services, their effectiveness^18^^,^^58^^,^^62^^,^^67^^,^^68^ , efficiency^18^^,^^58^^,^^62^^,^^67^^,^^68^, and capability to respond quickly^69^ were all noted as important. A specific concern was that infrastructure must have the capacity to deal with disaster^14^^,^^20^^,^^57^ and provide capable responses while in the midst of the crisis^70^. For example, the infrastructure must have processes in place to handle incoming information about a disaster and send instructions and implement a response during and after the disaster^13^^,^^31^^,^^71^.

In terms of public involvement and support, having local participation and representation in strategic planning, response, and recovery were described as important by multiple publications^13^^,^^19^^,^^22^^,^^48^^,^^52^^,^^72^. Additionally, public involvement may involve having local leaders who understand and represent a community’s uniqueness and aspirations. A sense of community empowerment can be an additional output of public involvement in governance and leadership^24^, as can increased trust in risk and crisis communication stemming from local leaders.

**Resources: **Numerous resources have been hypothesised to be connected with community resilience. From tangible supplies, such as food, water and first aid kits, to technical resources such as shelter, automobiles and essential machinery, a higher level of resources is generally supposed to lead to higher levels of resilience. Some publications have described ‘resources’ more generally as including intangible aspects such as “*natural, physical, human, financial, and social resources* (p. 2)"^31^ and suggested the importance of having these resources widely available and distributed in the community^30^^,^^48^^,^^69^. Ensuring the fairness of resource allocation is also known as distributive justice^73^. Additionally, for physical resources such as food or water, it has been suggested that merely possessing the resource is insufficient; a resilient community must be able to harness these resources^57^ and allocate them appropriately within the community^18^.

**Economic investment: **If not addressed, the direct and indirect economic costs of a disaster can plague an affected community long after it has occurred. Addressing the post-disaster economic situation can involve: (i) distribution of financial resources^48^, (ii) *economic programming* and ensuring that interventions are cost-effective^18^, and (iii) the* economic development* of the post-disaster infrastructure and increasing the diversity of economic resources^65^. This can be achieved through proactive investments to rebuild the economy^74^. Assessing a community’s current economy and developing its ability to sustain economic growth were also noted as important areas of concentration after a disaster^67^. The papers fell short in identifying a consensus for a useful post-disaster economic policy framework but many examples did overlap with suggestions provided by Smith^75^ in his book Planning for Post-Disaster Recovery: A Review of the United States Disaster Assistance Framework. The publications found in this review varied in terms of what economic planning is needed for a community in the pre-disaster setting, but all focused on the specific needs for post-disaster setting, whether through a specific programme to revitalise the job market, distribute economic aid or stimulate economic growth. A community’s post-disaster economy may be vital not just for recovery, but also for mitigating future disaster risks.

**Preparedness: **Almost all publications mentioned the importance of preparedness across a number of levels, including the individual, family and government. In spite of this, only a few publications identified specific preparedness activities that can be used to mitigate the effects of a disaster. For example, Tierney and Bruneau^68^ suggested that emergency management systems should create plans before a disaster on how the disaster-response processes would work. Similarly, risk assessment was believed to help with preparedness^61^. Actively involving community stakeholders in planning before a disaster, and running practice drills or exercises with a focus on risk management were cited as contributing to community resilience^22^^,^^24^. Carlson and colleagues^69^ suggested mitigation measures such as relocating buildings and infrastructure from flood-prone areas and/or flood-proofing structures, prior to a disaster. Altogether, the outputs of the planning, mitigation measures, and overall preparedness were intended to enable a sustainable response and recovery by the community, and to reduce the likelihood of harm to community members.

**Mental Outlook: **Mental outlook was defined as attitudes, feelings and views when facing the uncertainty that typically occurs after a disaster or when contemplating a future one. This term was conceptually different to mental health, as the latter dealt with well-being while the former dealt with attitudes towards uncertainty. After a disaster, uncertainty is a common feeling among the affected population. This uncertainty can manifest itself in different ways; from anxiety about what the future holds for families, to concerns about the long-term impacts on the community, uncertainty reaches across individual and group boundaries. The search for meaning and the quality of the meaning attached to the disaster can also affect a community’s outlook. The mental outlook of a community is therefore important in shaping the willingness and ability of community members to continue on in the face of uncertainty. For example, *hope*, the expectation that things will improve, can help people cope with the uncertainty caused by a disaster. Ganor and Ben-Lavy^64^ described hope as a vision of community that depicts a better future after a disaster. In addition to hope, *adaptability* can be defined as the ability and willingness to change after a disaster while accepting that things will be different. Many publications noted various aspects of adaptability as an inherent aspect of resilience (e.g. 8^,^^14^^,^^29^^,^^41^^,^^53^^,^^63^^,^^76^^,^^77^^,^^78^^,^^79^^,^^80^^,^^81^^,^^82^^,^^83^^,^^84^). Bahadur and colleagues^62^ argued that one of ten main characteristics of a resilient system is the “*acceptance of uncertainty and change* (p. 15).”

## Discussion

Within the field of disaster preparedness and response, it has been suggested that “*a required paradigm shift and a new national ‘culture of disaster resilience* (p. 2)"^74^ needs to occur. Unfortunately, our review suggests that we currently have no consensus on what such a culture would look like within our communities. What, exactly, do we mean by community resilience? Until we resolve this basic question, attempts to measure or enhance resilience will remain discordant and inefficient, while the academic literature will continue to be confused by papers assessing different concepts but using the same terminology.

At present, definitions within this field tend to either focus on specific aspects of the concept that may lead to overconfidence in the resilience of a given community that is deficient in elements that were not considered, or else tend towards all-encompassing definitions that may be too complex to apply at the local level. How can we advance from this apparent Catch-22? One option is to abandon the search for a single, precise definition of community resilience. Instead, it may be more appropriate to consider community resilience as a catch-all term for the range of elements which may be important for a community facing or recovering from a disaster. We are not alone in suggesting this. For example, Usher-Pines and colleagues^29^ point out, “*these discussions* [about the definition of community resilience]*, while important, distract stakeholders from the actual task at hand: to better prepare communities to respond and recover from incidents* (p. 604).” They argue that despite the advantages of the term community resilience, such as its ability to inspire people and re-invigorate the field, the pitfalls of community resilience are that no entity is clearly accountable for it and it is difficult to measure. A recent publication on subjective understandings of resilience-oriented interventions suggests similar ambiguity exists with the concept of ‘resilience'^85^. The authors argue that this ambiguity does not make the term meaningless but that researchers and professionals should understand that a diverse range of meanings exist, especially when considering interventions^85^. Rather than use the term community resilience, we therefore propose that it may be easier, clearer and more useful for academics, policy-makers and responders to be explicit as to the particular elements of resilience they are focussing on in their research or interventions; the phrase community resilience is not precise enough to be useful in any detailed discussion of the issue.

Our review identified an array of elements that have been proposed within the general notion of community resilience and that may be usefully explored further. *Factual knowledge base*, *collective efficacy and empowerment*, and *training and education* have been proposed as useful within the element of **local knowledge** in order to mitigate vulnerabilities caused by how a community understands its risks. The positive effects of *connectedness* and *cohesion* within the element of **community networks and relationships** have been seen, especially in recent times, to help people deal with uncertainty after a disaster. *Effective communication*, whether *risk* or *crisis* *communication*, was proposed as important in helping a community to articulate, coordinate and understand the risk and impact of disasters. *Health services* were clearly relevant for a disaster-affected community, though a lack of knowledge of a community’s pre-existing issues among its residents and/or difficulty in delivery of quick, high-quality care were identified as key areas of difficulty to guard against. Within the element of **governance and leadership**, ensuring that roles, participation/engagement, and front-line leadership during a crisis are clear at the local level appears to be the main emphasis in the current literature. Similarly, the fair distribution of **resources** may help communities in the short term, while **economic investment** was generally seen as a longer-term intervention to promote resilience. **Preparedness** overlapped with the elements of **local knowledge **and **communication** but was typified by an emphasis on specific actionable activities. Lastly, **mental outlook** arguably has the most potential to build resilience within a community through a focus on sub-elements such as *hope* and *adaptability*.

Most of these elements are already well-known within the disaster preparedness and crisis management fields outside of the specific rhetoric of community resilience; for example, risk and crisis communication has been extensively studied in respect to its role in disaster preparedness^86^^,^^87^^,^^88^. However, many of these elements are broad, overlapping in practice and need further clarification. For example, what specific economic processes are important in making a community resilient? What forms of social networks help in mitigating the effects of a disaster? What types of preparedness activities are most effective? Further progress on these and other questions might be best met by disaggregating the issues from the global concept of community resilience.

**Limitations: **Our review has several possible limitations. First, confirmation bias could have occurred when identifying publications for review given that a single researcher chose the accepted papers. To guard against this, we established an explicit set of inclusion criteria to use.

Second, it is possible to question the reliability of the thematic analysis of the review, given that only one researcher worked on it. Had someone else analysed the same data, they may have come to a different set of conclusions.

Third, selection bias based on language could have occurred, as there could have been additional useful studies available in languages other than English. Whether other elements are relevant in the resilience of non English-speaking communities is unknown. Whether community resilience is conceptualised differently in other cultures is an interesting question that may benefit from further investigation.

Fourth, it is unlikely that we identified every relevant study in the literature, especially the grey literature. Despite this, updating the literature search did not alter the fundamental structure of our results although it did add more examples for the main elements found in the review. This provides some reassurance that the inclusion of missing studies would not radically alter the nature of the elements that we identified.

Fifth, not all authors included in our review set out to write an original or specific definition of community resilience. For some of the included publications, community resilience was briefly described as part of another theory or concept. Had the authors of these papers been asked to construct their own formal definition, they may have produced a more detailed or nuanced interpretation. In many ways, however, the definitions given in these papers are of more interest, as they represent the interpretations of the concept that are being used in practice in the literature.

Sixth, the results of this review are based on the original authors’ definitions which were broken down and grouped by similar concepts through an inductive thematic analysis. This led us to identify nine elements. Further investigation is needed in order to determine whether these identified elements are attributes or processes which make a community resilient to a disaster. Possible determinants in classifying the elements might include the type of disaster, a community’s culture, and whether the element is measured before, during or after a disaster.

## Conclusion

The concept of community resilience is widely used in the academic and policy literature. Yet the meanings of the term differ from team to team. Nine core elements have been consistently suggested as constituting community resilience as it applies to disasters: local knowledge, community networks and relationships, communication, health, governance and leadership, resources, economic investment, preparedness, and mental outlook. Further exploration of these individual elements may lead to a greater understanding of what community resilience is and how it can be measured and enhanced. In the meantime, the use of the phrase community resilience, and attempts to define the concept, may be unhelpful if it obscures the importance of these individual elements.

## Competing Interest Statement

The authors have no competing interests.

## Corresponding Authors

Sonny S. Patel (spatel@hsph.harvard.edu)

G. James Rubin (gideon.rubin@kcl.ac.uk)

## Data Availability Statement

All relevant data are within the article.

## Appendices

**Supplementary Material Table 1. Defining community resilience (resilience in community setting) in regards to a disaster.** 

Reference(s)	Year(s) of Publication(s)	Definition(s) (direct quote)
(89)	1998	It is proposed that mediating structures (e.g., schools, peer groups, family) and activity settings moderate the impact of oppressive systems and provide contexts for resilience and consciousness raising (pg. 460)
(72)	2000	A resilient community is one that takes intentional action to enhance the personal and collective capacity of its citizens and institutions to respond to, and influence the course of social and economic change (pg. 1-5)
(53)	2001	The personal and community characteristics and processes that promote a capability to “bounce back” and to use physical and economic resources effectively to aid recovery following exposure to hazard activity. [inferred as CR] (pg. 158)
(90)	2003	Community seismic resilience is defined as the ability of social units (e.g., organizations, communities) to mitigate hazards, contain the effects of disasters when they occur, and carry out recovery activities in ways that minimize social disruption and mitigate the effects of future earthquakes. (pg. 735)
(64)	2003	Community resilience is the ability of a community to stick together and to help itself as a group, as well as the families and individuals in its midst. (pg. 106)
(83)	2003	In the context of hazards, the concept spans both pre-event measures that seek to prevent disaster-related damage and post-event strategies designed to cope with and minimize disaster impacts (pg. 3)
(58)	2004	We define community resilience as including those features of a community that in general promote the safety of its residents and serve as a specific buffer against injury and violence risks, and more generally, adversity. (pg. 391)
(25, 27)	2004 and 2007	Resilience to loss and trauma pertains to the ability of adults in otherwise normal circumstances who are exposed to an isolated and potentially highly disruptive event, such as the death of a close relation or a violent or life-threatening situation, to maintain relatively stable, healthy levels of psychological and physical functioning... as well as the capacity for generative experiences and positive emotions (pgs. 20-21)
(18)	2004	Effective recovery can be achieved only where the affected community participates fully in the recovery process and where it has the capacity, skills and knowledge to make its participation meaningful (pg.6) A community’s capacities, skills, and knowledge that allow it to participate fully in recovery from disasters [inferred by (52) on p. 129]
(13)	2004	Community resilience refers to “the capacity of a human community, whether a city, a region, or some other collectivity, to sustain itself through crises that challenge its physical environment and social fabric” (pg. 64)
(91)	2004	Thus, community resilience is defined in this paper as individuals’ sense of the ability of their own community to deal successfully with the ongoing political violence. (pg. 442)
(51)	2005	The construct of resilience can be defined as a process or the attainment of positive outcomes at the individual, family, and community levels despite adversity (e.g., natural disaster, terrorist attack) (pg. 319)
(21)	2005	Recovery is defined as ‘decisions and actions taken after a disaster with a view to restoring or improving the pre-disaster living conditions of the stricken community, while encouraging and facilitating necessary adjustments to reduce disaster risk’ (pg. 33)
(79)	2006	Disaster resilience could be viewed as the intrinsic capacity of a system, community or society predisposed to a shock or stress to adapt and survive by changing its non-essential attributes and rebuilding itself (pg. 443)
(92)	2007	The ability of community members to take meaningful, deliberate, collective action to remedy the impact of a problem, including the ability to interpret the environment, intervene, and move on. More than the ability of members to cope individually, community resilience involves interactions as a collective unit. (pg. 349)
(68)	2007	Disaster resilience as the ability of social units (e.g., organizations, communities) to mitigate hazards, contain the effects of disasters when they occur, and carry out recovery activities in ways that minimize social disruption and mitigate the effects of future disasters (pg. 15) Resilience can be measured by the functionality of an infrastructure system after a disaster and also by the time it takes for a system to return to pre-disaster levels of performance (pg. 15)
(20)	2007	System or community resilience can be understood as: capacity to absorb stress or destructive forces through resistance or adaptation, capacity to manage, or maintain certain basic functions and structures, during disastrous events, and capacity to recover or ‘bounce back’ after an event (pg. 6)
(93)	2008	Resilience - the ability to go through trauma and to introject meaning into one's own life (pg. 3)
(8, 63)	2008	Resilience is the ability of a social system to respond and recover from disasters and includes those inherent conditions that allow the system to absorb impacts and cope with the event, as well as post-event adaptive processes that facilitate the ability of the system to re-organize, change, and learn in response to the event (pg. 599)
(52)	2008	A process linking a set of networked adaptive capacities to a positive trajectory of functioning and adaptation in a constituent populations after a disturbance (pg. 131)
(78)	2009	A new body of work is attempting to expand the focus on resilience as a characteristic of the individual to one of resilience as a community and cultural process. This new focus on “community resilience” looks at how people overcome stress, trauma and other life challenges by drawing from the social and cultural networks and practices that constitute communities. At the same time, it draws attention to the resilience of the community itself. (pg. 63)
(62)	2010	[Provides various definitions across disciplines and offer 10 defining characteristics of resilient systems as follows]1) a high level of diversity in community 2) effective governance and institutions which may enhance community cohesion 3) the inevitable existence of uncertainty and change is accepted 4) there is community involvement and the appropriation of local knowledge in any resilience-building projects; communities enjoy ownership of natural resources; communities have a voice in relevant policy processes 5) preparedness activities aim not at resisting change but preparing to live with it 6) high degree of social and economic equity exists in systems 7) importance of social values and structures is acknowledged because association between individuals can have a positive impact on cooperation in a community which may lead to more equal access to natural resources and greater resilience 8) non-equilibrium dynamics of a system are acknowledged and building resilience should not work with idea of restoring equilibrium 9) continual and effective learning is important 10) resilience systems take a cross-scalar perspective of events and occurrences. Resilience is built through social, political, economic and cultural networks that reach from the local to the global scale (pgs. 2-3)
(94)	2010	We also propose a more expanded definition of community that explicitly includes the resources, social links, and social climate of state and federal actors in relation to the community. Disasters often overwhelm the local community’s ability to respond, and modifying the framework as we propose recognizes the need for considering resources such as the National Guard, the VHA, and the social climate of the country as it observes the impact of a disaster on television [Inferred definition of community resilience] (pg. 585)
(95)	2010	The capacity of a system to absorb disturbance and reorganize while undergoing change so to still retain essentially the same function, structure and feedbacks, and therefore identity, that is , the capacity to change in order to maintain the same identity (pg. 3)
(56)	2010	Resilience is not a process, it is not a management system standard, nor is it a consulting product. Resilience is a demonstrable outcome of an organization’s capability to cope with uncertainty and change in an often volatile environment. Resilience is thus a product of an organization’s capabilities interacting with its environment (pg. 246)
(57)	2010	Communities and individuals harnessing local resources and expertise to help themselves in an emergency, in a way that complements the response of the emergency services (pg. 4)
(17)	2010	Resilience is not an end state but a dynamic process of interdependent forces - at the individual, family, group, and community levels - that continually shape and reshape the organism (pgs. 268-269). Community resilience consists of both reactive and proactive elements that join recovery from adversity with individual and group efforts to transform their environments to mitigate future problems or events. Thus, community resilience is not simply the return to homeostasis, but rather implies a potential to group from adversity that derives, in part, from deliberate, meaningful cooperation and action (pg. 269).
(67)	2010	Resilience may be defined as a function indicating the capability to sustain a level of functionality or performance for a given building, bridge, lifeline network, or community, over a period defined as the control time (pg. 2)
(55)	2011	The capability (or process) of a community adapting and functioning in the face of disturbance (pg. 370)
(22)	2011	Community resilience entails the ongoing and developing capacity of the community to account for its vulnerabilities and develop capabilities that aid that community in (1) preventing, withstanding, and mitigating the stress of a health incident; (2) recovering in a way that restores the community to a state of self-sufficiency and at least the same level of health and social functioning after a health incident; and (3) using knowledge from a past response to strengthen the community’s ability to withstand the next health incident (pg. 9)
(96)	2011	Rather than define disaster resilience, the Strategy focuses on the common characteristics of disaster resilient communities, individuals and organisations. These characteristics are functioning well while under stress, successful adaptation, self-reliance, and social capacity...Resilient communities also share the importance of social support systems, such as neighbourhoods, family and kinship networks, social cohesion, mutual interest groups, and mutual self-help groups. (pg. 4)
(54)	2011	The construct of resilience is generally understood as the capability of a community to face a threat, survive and bounce back or, perhaps more accurately, bounce forward into a normalcy newly defined by the disaster related losses and changes. Community resilience is, in effect, a reflection of people’s shared and unique capacities to manage and adaptively respond to the extraordinary demands on resources and the losses associated with disasters (pg. 396)
(14)	2011	Disaster resilience is the ability of countries, communities and households to manage change, by maintaining or transforming living standards in the face of shows or stresses - such as earthquakes, drought or violent conflict - without compromising their long-term prospects. (pg. 6)
(31, 71)	2011 and 2013	Resilience can be thought of as attribute (an ability or capacity), a process, and/or an outcome associated with successful adaption to, and recovery from adversity. Building a resilient community involves more than assembling a collection of resilient individuals. Community resilience requires that the community as a whole must cope effectively with and learn from adversity. A resilience community has the ability to transform the environment through deliberate, collective action. (pg. 1) Community resilience entails the ability of community members to take deliberate, purposeful, and collective action to alleviate the detrimental effects of adverse events. (pg. 251)
(97)	2011	The characteristics of resilient communities identified in the literature incorporate core dimensions of social capital: such as the centrality of networks and social relationships (connections for groups to work collaboratively) and norms of trust and reciprocity (essential for networks and collaboration to exist). (pg. 6) Resilient communities are those with well-developed networks and strong social relations as well as norms of trust and reciprocity [Inferred from (98) on pg. 470]
(99)	2011	‘Resilience’ is a relative term that can look wildly different in different contexts and according to different developmental stages of community life. Likewise, ‘community’ is a contested idea that makes different kinds of sense according to the value, location and perspective of the reader (pg. 4)
(100)	2012	Beyond the resilience of individuals or individual organisations, your community will prove resilient in the event of a severe emergency or disaster when members of the population are connected to one another and work together, so that they are able to: 1) function and sustain critical systems, even under stress; 2) adapt to changes in the physical, social or economic environment; 3) be self-reliant if external resources are limited or cut off; 4) learn from experience to improve over time (pg. 17)
(69)	2012	The ability of an entity - asset, organization, community, region - to anticipate, resist, absorb, respond to, adapt to, and recover from a disturbance (pg. 17) Community/regional resilience is a function of the resilience of several subsystems, including but not necessarily limited to, the community/region’s economy, civil society, critical infrastructure, supply chains/dependencies, and governance (including emergency services) (pg.viii).
(74)	2012	Resilience: the ability to prepare and plan for, absorb, recover from or more successfully adapt to actual or potential adverse events (pg. 14)
(41)	2012	Resilience can be defined as capacity of a dynamic system to withstand or recover from significant challenges that threaten its stability, viability, or development (pg. 231)
(101)	2012	Reducing disaster losses and restoring the life of communities are essential to any meaningful definition of sustainability. The capacity to speed recovery by taking action in advance to identify and reduce vulnerabilities is known as resilience (pg. 41)
(24)	2012	This report focuses on CR [community resilience] as the ability of a community to fortify itself so that it is able to prevent, respond to, and recover from a natural or intentional public health disaster (pg. 292)
(80)	2012	Within preparedness phase, resistance is defined as the ability of an individual, a group, an organization, or even an entire population to withstand manifestations of clinical distress, impairment or dysfunction associated with critical incidents, terrorism, and disasters. (pg. 73) Within immediate post event phase, resilience is defined as the ability of an individual, a group, an organization, or even an entire population to rapidly and effectively rebound from psychological perturbations associated with critical incidents, terrorism, and disasters. (pgs. 73-74) For the population who have not bounced back and continue to have problems well after the disaster event, recovery is defined as the ability of an individual, a group, an organization, or even an entire population to restore their adaptability and function, both psychologically and behaviourally, in the wake of significant clinical distress, impairment, or dysfunction subsequent to critical incidents such terrorism, acts of violence and disasters. (pg. 74)
(76)	2012	Disaster Risk Reduction is the concept and practice of reducing disaster risks through systematic efforts to analyse and manage the causal factors of disasters, including through reduced exposure to hazards, lessened vulnerability of people and property, wise management of land and the environment, and improved preparedness for adverse events. (pg. 3) Resilience is the ability of a system, community or society exposed to hazards to resist, absorb, accommodate to and recover from the effects of a hazard in a timely and efficient manner, including through the preservation and restoration of its essential basic structures and functions. (pg. 3)
(48)	2012	Resilience is defined as adaptation and coping despite collective adversity in a system (individual, family, organization, community, country). (pg. 147)
(102)	2013	Resilient people conceptualise the world as being organized understandable and prevents them from developing symptoms of trauma (pgs. 1-2)
(103, 104)	2013	A community’s ability to rebound to a healthy state following a major disruption such as a disaster (pg. 2). As applied to disasters, resilience entails the ability of a community to rebound following a hurricane, earthquake, or other disturbance (pg. 1)
(23)	2013	The National Policy enthusiasm for re-envisioning the preparedness agenda around community resilience (the ability to prevent, withstand, and mitigate the stress of a disaster) raises questions among local health departments (LHDs) about how to build or strengthen community resilience and how to integrate the “whole of community approach (a community-integrated model to involve a diverse set of stakeholders) in usual disaster-planning activities. (pg. 1181)
(32, 105)	2013	The term Community Resilience is used to describe the community’s ability to deal with crises or disruptions. (pg. 1732) In present study we refer to Community resilience as the community’s ability to withstand crises or disruptions (pg. 314)
(77)	2013	Community resilience is the capability to anticipate risk, limit impact, and bounce back rapidly through survival, adaptability, evolution, and growth in the face of turbulent change (pg. 14)
(106)	2013	Community resilience refers to the capacity or ability of a community to anticipate risk, prepare for, respond to and recover rapidly through survival, adaptability, evolution and growth from experiencing disasters and their impacts (pg.4)
(107)	2013	The capacity of a community to change and develop following the challenge (pg. 263); involves empowering the local informal and formal leadership and training citizens in neighbourhoods and institutions, including schools, regarding home and institutional preparation, medical and psychological first aid and community and family resilience (pg. 268)
(12)	2013	Disaster mitigation beings long before impact and is defined as the actions taken by a community to eliminate or minimize the impact of a disaster...The resilience of a community overwhelmed by a disastrous situation may be measured in the difference between a response with a sense of hope, community pride, and resourcefulness and one filled with despair, hopelessness, and blame. A community’s assessing vulnerabilities, developing resilient infrastructure, establishing memoranda of understanding, and planning for a sustainable response leads to mitigation of an event long before the actual impact. (pg. 13)
(81)	2013	The sustained ability of a community to withstand and recover from adversity (e.g., economic stress, pandemic influenza, manmade or natural disasters) (pg. 1191)
(15)	2013	For clarity purposes, here we adopted the same definition as the Intergovernmental Panel on Climate Change, which describes resilience as “the ability of a system and its component parts to anticipate, absorb, accommodate, or recover from the effects of a hazardous event in a timely and efficient manner, including through ensuring the preservation, restoration, or improvement of its essential basic structures and functions” (108). (pg. 1)
(109)	2013	Resilience has been defined in many ways but they all refer to the capacity of a community to assess its risks, needs, resources, and skills accurately, and to reallocate resources and attention to meet changing demands with timely action. (pg. 159)
(19)	2013	Resilience refers to the capacity of an individual, household, population group or system to anticipate, absorb, and recover from hazards and/or effects of climate change and other shocks and stresses without compromising (and potentially enhancing) long-term prospects. (pg. 9)
(29)	2013	Ultimate vision of CR [community resilience]: communities that are able to withstand and recover from adversity. (pg. 605)
(110)	2013	Building capacity for CDR [community disaster resilience] requires an approach suitable to integrating and coordinating the perspectives and skills of diverse stakeholders, including historically vulnerable groups, first responders, and experts in evidence-based approaches to improving outcomes, including for mental health consequences of disasters. (pg. 452)
(84)	2013	The ability of a system and its component parts to anticipate, absorb, accommodate or recover from the effects of a hazardous event in a timely and efficient manner, including through ensuring the reservation, restoration or improvement of its essential basic structures and functions. (pg. 4)
(111)	2014	Community Resilience can be understood as the capacity of a system, community or society to adapt in the face of hazards by taking action in order to reach and maintain an acceptable level of function and structure. In part, this is determined by how much a community is capable of self-organisation to maximise risk reduction measures and apply learning from past disasters to forward-looking disaster preparedness. (pg. 1)
(70)	2015	The foundation of the Resilience Activation Framework (RAF) is grounded in distinguishing resilience processes (the ability to withstand, adapt, or recover quickly from a disaster), individual and community resilience attributes, and the factors which facilitate the activation of those resilience attributes. (pg. 43) Community resilience can be defined as the enduring capacity of geographically, politically, or affinity-bound communities to define and account for their vulnerabilities to disaster and develop capabilities to prevent, withstand, or mitigate for a traumatic event [cited (22, 52)]. (pg. 48)
(16)	2015	Community resilience describes the collective ability of a neighbourhood or geographically defined area to deal with stressors and efficiently resume the rhythms of daily life through cooperation following shocks. (pg.255)
(61)	2015	Community disaster resilience (CDR)-the ability of a community to survive and thrive in the face of uncertainty-is the foundation of rural life. [same definition as (54)] (pg. 220)
(65)	2015	Overall, as a process, community resilience is not an outcome. Rather, community resilience is indicated by evidence of community well-being fooling a disaster or crisis. Thus community provides an opportunity for a collective to adaptively cope with the experience of a potentially traumatic event. (pg. 279)
(66)	2015	Resilience, defined as a trajectory of low levels of symptoms or problems in a given outcome over time, with minimal elevations that are limited to the time period during the disaster and its immediate aftermath...We differentiate between this general definition of wellness, which we labelgeneral wellness, andmental health wellness, which we define as resilience across various conditions within the mental health domain (e.g. PTSS and depression) specifically (pg. 162)
(112)	2015	Psychological resilience, defined as the ability to “bounce back” from disaster, sustaining low levels of psychological symptoms over time...Given the proposed interdependence of resilience at multiple levels, it is likely that community-level resources and exposure exert direct effects on individual-level psychological resilience, as well as influence the relationship between individual-level disaster exposure and resilience....Far less attention has been paid to the characteristics of communities that influence responses. This is an important limitation given that the resilience of individuals is inextricably linked to the resilience of the communities in which they live (pg. 2)
(59)	2015	Ability of a system, community, or society exposed to hazards to resist, absorb, accommodate to and recover from the effects of a hazard in a timely and efficient manner including through the preservation and restoration of its essential basic structures and functions [same definition as (9)](pg. 3)
(60)	2015	Resilience can be defined as an attribute (e.g., ability, capacity), a process, and/or an outcome associated with successful adaption to, and recovery from adversity. Definitions differ depending on context and purpose. (pg. 241)
(113)	2015	As described in the current online CART manual, the CART Assessment Survey is based on a four-factor model of community resilience characterized by four interrelated CART domains: (a) Connection and Caring (including relatedness, participation, shared values, support and nurturance, equity, justice, hope, and diversity); (b) Resources (including natural, physical, information, human, social, and financial resources); (c) Transformative Potential (deriving from the ability of communities to frame collective experiences, collect and analyse relevant data, assess community performance, and build skills); and (d) Disaster Management (addressing prevention and mitigation, preparedness, response, and recovery). Recognizing the importance of information and communication in community resilience, one goal of the current study was to confirm the existence of a fifth domain, Information and Communication, addressing the availability of information and trust in public officials. (pg. 182)
(82)	2015	We hypothesize resilience as a protective process with the capacity for orienting and leading coping abilities toward a successful outcome in a population exposed to a natural disaster. (pg. 56)
(30)	2015	Resilience describes attributes and capabilities that enable an entity to dynamically adjust and positively adapt to adverse forces or impacts and emerge afterward in a positive functional state. (pgs. 201-202)

**Supplementary Material Table 2. The Constituent Elements of Community Resilience:** 

Elements	Specific sub-elements with references
Local knowledge	Factual knowledge base(e.g. knowledge of treatment (58); lessons from past disasters (111); knowledge of basic life support techniques (55); risk knowledge (59); assessment or accountability of vulnerabilities (12, 70, 101); knowledge of expertise (57); response (17, 31, 60, 92, 113); knowledge and skills translatable to disaster preparedness, mitigation, response, recovery (61); disaster and risk information (65); integrated knowledge base (109))Collective efficacy and empowerment(e.g. knowledge of self-reliance, self-help, and self-sufficiency (22); self-reliance (96); recovery mechanisms (69); sense of coherence (102); capacity for generative experiences and positive emotions (25, 27); self-efficacy (80); community competence, collective efficacy, empowerment (52, 66); individual believes that s/he has the resources needed to deal with situations (102))Training and education(e.g. knowledge and education (20, 61, 70); community education and training(22-24, 70); continual and effective learning (62); learn in response to the event (8, 63); knowledge transfer (19); risk awareness training (111); social, lifestyle and community competence (54, 59, 67, 82); learning (education, knowledge, risk awareness based on previous experience) (55); practice (24))
Community networks and relationships	Connectedness(e.g. community networks and relationships(16, 58); connectedness (17, 31, 60, 92, 113); social networks and capital (16, 54, 61, 103, 104); self-sufficiency (22, 23); social or cross-community links(94, 99); social relationships (91, 97); connection and caring (71, 113); social and/or cultural networks (24, 61, 78); social and cultural capital (59); partnership and activating networks (110); neighbourhoods, family and kinship networks (8, 63, 89))Cohesion(e.g. social capacity, social support systems, and social cohesion (8, 63, 96); collective social support and collective efficacy (66); social support (15); social capital (97, 112); community cohesion (62, 64, 70); social connectedness (22, 23); social capital (specified to include trust and social cohesion) (16, 52, 54, 55, 61, 70); build trust and strong ties (13, 16, 97); social networks and capital (103, 104); commitment and shared values (17, 31, 92, 113); collective cohesion and self-efficacy (16, 70); community pride (12))
Communication	Risk communication(e.g. risk communication (22, 23, 57); information resilience (107); risk awareness (55); public risk communications (24); risk information and awareness (65); communications infrastructure and technology (14); coordinated communication system or network (17))Crisis communication(e.g. coordinate effectively during response (55, 65); open communication during crisis (13); flow of information in real time (64); communications infrastructure and technology (61); coordinated communication system or network (22))Effective communication(e.g. memoranda of understanding (12); information and communication (creation of common meanings and understandings and the provision of opportunities for members to articulate needs, views, and attitudes) (52, 113); communication (17, 19, 31, 92, 110); strong communication networks (22))
Health	Health services(e.g. healthcare services(21); medical and mental health services (107); health and mental health resilience (107); healthcare and related social services (22); medical care (70); emergency response capabilities (61)):Physical health(e.g. physical health (15, 22, 23, 55); physical functioning (25, 27))Mental health(e.g. psychological health (22, 23); psychological functioning (25, 27); mental health (15, 55); coping (64); mental health wellness, trauma, PTSS and depression (66); PTSD (112); clinical distress (80))
Governance and leadership	Infrastructure and services(e.g. good governance (18); infrastructure (21); organized governmental services (59, 67); analyse and manage the causal factors of disasters(76); presence of community structures and leadership (58); effective governance and institutions (62); ensuring the reservation, restoration or improvement of essential basic structures and functions (84); disaster management (71, 113); leadership (32, 91, 105); leadership that improve disaster-related organizational performance and problem solving(68); response capabilities (69); capacity to respond and recover its full range of functions (13); function and sustain critical systems, even under stress (100); disaster preparedness and response (20); global response (57); capacity to deal with disturbance (14); development of infrastructure (12); capable governance (70); disaster-related exposure, such as buildings affected (112); leading coping abilities toward a successful outcome (82))Public involvement and support(e.g. community leadership and mutual support (18); partnership and engagement (22, 23); public involvement (13); community engagement (61, 110); credibility (leadership represent community’s uniqueness and aspirations) (64); structure, roles, and responsibilities (17, 31, 92, 113); self-organisation to maximise risk reduction measures(111); leadership, participation, and representation (19); community process (strategic thinking, participation and action) (72); community empowerment (24); level of social integration of government and nongovernmental organizations in planning, response, and recovery(22, 94); active engagement of community stakeholders in health event planning and personal preparedness (22); collective action and decision-making(52); collective action (48); decision support system (109))
Resources	Resources(e.g. adequate resourcing (18, 109); harnessing local resources (57); awareness and use of resources in the community (72); available resources (100); community-level resources (112); resource management (61); distribution of tangible resources (48 , 69, 70); social resources (70, 91, 94); resources (15, 17, 19, 31, 92, 113); natural and economic resources (61); access to money and other financial instruments and assets (70); diverse economic resources (65); physical infrastructure (59); food services and distribution (30); physical and economic resources effectively used to aid recovery (53))
Economic investment	Post-disaster economic development(e.g. economic capacity and diversification (55); economic equity (62, 81); economic development (52, 59, 112); economic well-being (22, 23); economic development (61, 65, 67); localised economy within ecological limits (99); economic rehabilitation (21); proactive investments and policy decisions to reduce loss of lives, costs, and socioeconomic impacts of future disasters (74); economic resilience (69); access to money and other financial instruments and assets (70); diverse economic resources (65))Post-disaster economic programming(e.g. cost-effective programming (18); distribution of economic resources (48); employment-seeking behaviour (58); livelihood and economic development interventions (111); employment and occupational diversity (70); economic access for coping with disaster (8, 19, 63, 68, 83, 90); economic resilience(69); economy education (30))
Preparedness	Planning and mitigation(e.g. preparedness activities (32, 62, 69, 80, 105); planning and procedures(100); applying lessons learned from past disasters to forward-looking disaster preparedness (111); mitigation measures (69); planning for sustainable response and recovery (12); distribution of disaster mitigation (prevention) projects (48); disaster prevention and mitigation, preparedness and withstanding the disaster (12, 17, 23, 31, 32, 70, 74, 76, 92, 105, 113); hazards mitigation and planning (63); planning that improve disaster-related organizational performance and problem solving(68); disaster preparedness(20, 111); capacity to plan, buffer, and/or protect its full range of functions (13, 58); active engagement of community stakeholders in health event planning and personal preparedness (22); risk management practice (24); hazard risk assessment (12, 61); anticipating risk and limit impact (77, 84, 106))
Mental Outlook	Hope(e.g. hope and ability to persevere in spite of adversity (58); credo (vision of community that depicts a better future/ horizon of hope) (64); introject meaning into one’s own life (93); sense of hope and community pride (12))Adaptability(e.g. cope with or acceptance of uncertainty and change (56, 62); adaptability (14, 48, 55, 95, 106, 111); adaptive capacity (22, 52); reaction to disturbance (e.g. adapt, survive, cope, recover, learn, transform, bounce back) (8, 14, 29, 41, 53, 63, 76-84); transformability(95); individual believes that s/he has the resources needed to deal with situations (102); previous traumatic experiences (15); adapt to changes in the physical, social or economic environment(100); survival, evolution and growth from experience and impacts (106); positive outcomes despite adversity (30, 51); speed recovery (101))

**Supplementary Material Table 3. Comparison between Elements Found in Review to Widely Cited Reviews, Models and Measurements:** 

Elements/sub-elements	Resilience Review (55)	CDR Review(59)	RAND report(22)	DROP Model(8)	Community Resilience Index(52,114)	CCRAM (32,105)	CART (31,71)
Local knowledge	X	X	X	X	X		X
Factual knowledge base		X		X	X		
Collective efficacy and empowerment			X		X	X	X
Training and education	X		X	X	X		
Community networks and relationships	X	X	X	X	X	X	X
Connectedness			X	X	X		X
Cohesion	X		X	X	X		X
Communication	X		X	X	X		X
Risk Communication			X	X			
Crisis Communication			X				
Effective Communication			X		X		
Health			X	X	X		
Health services			X		X		
Physical Health	X		X				
Mental Health	X		X	X	X		X
Governance and leadership	X	X	X	X	X	X	X
Infrastructure and services		X		X	X		
Public involvement and support			X	X			X
Resources			X		X		X
Economic investment	X	X	X	X	X		X
Post-disaster economic development		X	X	X	X		
Post-disaster economic programming		X					
Preparedness	X		X	X	X	X	X
Planning and mitigation	X		X	X	X		X
Mental Outlook							
Hope					X		
Adaptability	X		X	X	X	X	X

## List of abbreviations

CART: Communities Advancing Resilience Toolkit

CCRAM: Conjoint Community Resiliency Assessment Measure

PTSD: Post-Traumatic Stress Disorder

PTSS: Post-traumatic Stress Syndrome

CR: Community Resilience

CDR: Community Disaster Resilience
